# Differences of Phenylalanine Concentrations in Dried Blood Spots and in Plasma: Erythrocytes as a Neglected Component for This Observation

**DOI:** 10.3390/metabo11100680

**Published:** 2021-10-03

**Authors:** Dorothea Haas, Jana Hauke, Kathrin V. Schwarz, Lucia Consalvi, Friedrich K. Trefz, Nenad Blau, Georg F. Hoffmann, Peter Burgard, Sven F. Garbade, Jürgen G. Okun

**Affiliations:** 1 Division of Child Neurology and Metabolic Medicine, Center for Child and Adolescent Medicine, University Hospital Heidelberg, 69120 Heidelberg, Germany; jana.hauke@med.uni-heidelberg.de (J.H.); kathrin.schwarz@med.uni-heidelberg.de (K.V.S.); lucia.consalvi@med.uni-heidelberg.de (L.C.); friedrich.trefz@metabolic-consulting.de (F.K.T.); georg.hoffmann@med.uni-heidelberg.de (G.F.H.); peter.burgard@t-online.de (P.B.); sven.garbade@med.uni-heidelberg.de (S.F.G.); juergenguenther.okun@med.uni-heidelberg.de (J.G.O.); 2 Division of Metabolism, University Children’s Hospital Zürich, 8032 Zürich, Switzerland; nenad.blau@kispi.uzh.ch

**Keywords:** phenylketonuria, PKU, phenylalanine, treatment, dried blood spot, plasma, measurement, monitoring

## Abstract

Monitoring phenylalanine (Phe) concentrations is critical for the management of phenylketonuria (PKU). This can be done in dried blood spots (DBS) or in EDTA plasma derived from capillary or venous blood. Different techniques are used to measure Phe, the most common being flow-injection analysis tandem mass spectrometry (FIA-MS-MS) and ion exchange chromatography (IEC). Significant differences have been reported between Phe concentrations in various sample types measured by different techniques, the cause of which is not yet understood. We measured Phe concentrations in 240 venous blood samples from 199 patients with hyperphenylalaninemia in dried blood spots, EDTA plasma and erythrocytes by FIA-MS-MS and IEC. Phe concentrations were significantly lower in erythrocytes than in plasma leading to about 19% lower Phe DBS concentrations compared with plasma independent from the method used for quantification. As most therapy recommendations for PKU patients are based on plasma concentrations reliable conversion of DBS into plasma concentrations is necessary. Variances of Phe concentrations in plasma and DBS are not linear but increases with higher concentrations indicating heteroscedasticity. We therefore suggest the slope of the 75th percentile from quantile regression as a correction factor.

## 1. Introduction

Phenylketonuria (PKU, OMIM 261600) is an autosomal recessive disorder of phenylalanine metabolism characterized by deficient phenylalanine hydroxylase (PAH, EC 1.14.16.1). PAH hydrolyzes the amino acid phenylalanine (Phe) to tyrosine (Tyr) using tetrahydrobiopterin (BH_4_) as a cofactor. Its deficiency caused by pathogenic variants in the *PAH* gene results in accumulation of Phe and a relative deficiency of Tyr leading to neurological abnormalities and irreversible cognitive impairment [1]. Early detection by newborn screening and prompt initiation of medical or dietary treatment prevents these devastating complications and ensures normal intellectual development [2]. However, Phe concentrations are influenced by nutrition and activity, but also by disease. Target ranges for specific age groups and for pregnancy have been established and regular monitoring and adaption of therapy according to Phe concentrations is mandatory. Current guidelines suggest twice weekly to monthly controls in infants and children, and monthly controls in adults, and dietary Phe intake is adjusted according to these results [3]. Controls are most frequently performed from dried blood spots (DBS) collected from capillary blood by finger prick at home and mailed to the laboratory as it is most convenient for the patients. However, Phe concentrations measured in DBS by flow-injection analysis tandem mass spectrometry (FIA-MS-MS) differ from those measured by ion exchange chromatography (IEC) in EDTA or lithium heparin plasma [4,5,6] with concentrations being 18 to 28% lower in DBS [7]. Most of the recommended target ranges refer to plasma Phe concentrations [8,9]. In order to follow these guidelines adequately, and to advise patients on treatment options, a reliable conversion from DBS to plasma Phe concentration is necessary.

In this study we compare Phe concentrations measured by IEC and FIA-MS-MS in different preparations from venous blood (erythrocytes, liquid and dried EDTA plasma and DBS). We show that Phe concentrations are significantly lower in erythrocytes explaining the difference of Phe in DBS compared to plasma. However, exact quantification of Phe concentration in erythrocytes is difficult as cells have to be washed before analysis to avoid contamination by plasma components. This procedure may lead to washout of Phe and other amino acids by saline solution [10]. We address this problem and propose a formula to convert Phe concentrations measured in DBS to those in plasma.

## 2. Results

Phe in the venous blood of 199 PKU patients was measured in four different specimens (liquid plasma, dried plasma, dried whole blood spots (DBS), washed erythrocytes spotted on a filter paper card) by two different methods (IEC, FIA-MS-MS). In all 240 samples, Phe was measured in liquid plasma by IEC, in dried plasma by FIA-MS-MS and in DBS by FIA-MS-MS. In a subset of 10 randomly chosen samples Phe was additionally measured in DBS by IEC and in erythrocytes by IEC and FIA-MS-MS. In 228 samples hematocrit was also determined.

### 2.1. Comparison of Methods (IEC and FIA-MS-MS)

**Figure 1:**
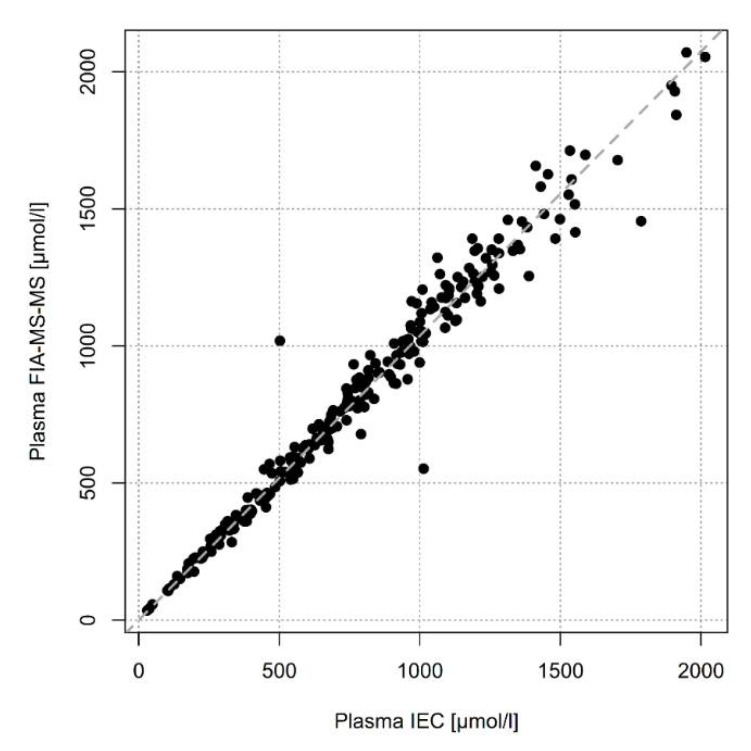
Regression of plasma Phe measured by FIA-MS-MS on plasma Phe measured by IEC (n = 240).

Quantification of Phe in liquid plasma measured by IEC and dried plasma measured by FIA-MS-MS in 240 samples showed similar concentrations. Concentrations were slightly higher when measurement was performed by FIA-MS-MS (y = 1.04x) with only minimal variance (R^2^ = 0.97) in a concentration range from 0 to 2000 µmol (Figure 1).

**Figure 2:**
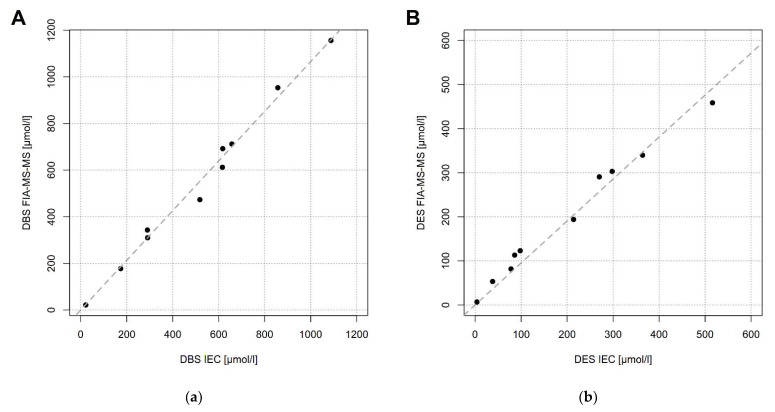
Comparison of Phe concentrations in dried blood (DBS) (**a**) and erythrocytes (DES) (**b**) measured by IEC (*x*-axis) and FIA-MS-MS (*y*-axis) (n = 10).

Next, we measured Phe concentrations in DBS by IEC and by FIA-MS-MS in 10 samples. There was only minimal difference with slightly higher concentrations when samples were measured by FIA-MS-MS (y = 1.07x). Variance was negligible (R^2^ = 0.99) in a concentration range from 0 to 1200 µmol/L (Figure 2a). Phe concentration in erythrocytes was 5% lower when measured by FIA-MS-MS (y = 0.95x) in a concentration range from 0 to 500 µmol/L. Variance of residuals was minimal (R^2^ = 0.97, Figure 2b).

Therefore, both methods (IEC and FIA-MS-MS) provide similar results in plasma and DBS. The differences between them are not significant and can be neglected.

### 2.2. Comparison of Phe Concentrations in Different Blood Compartments by One Method

**Figure 3:**
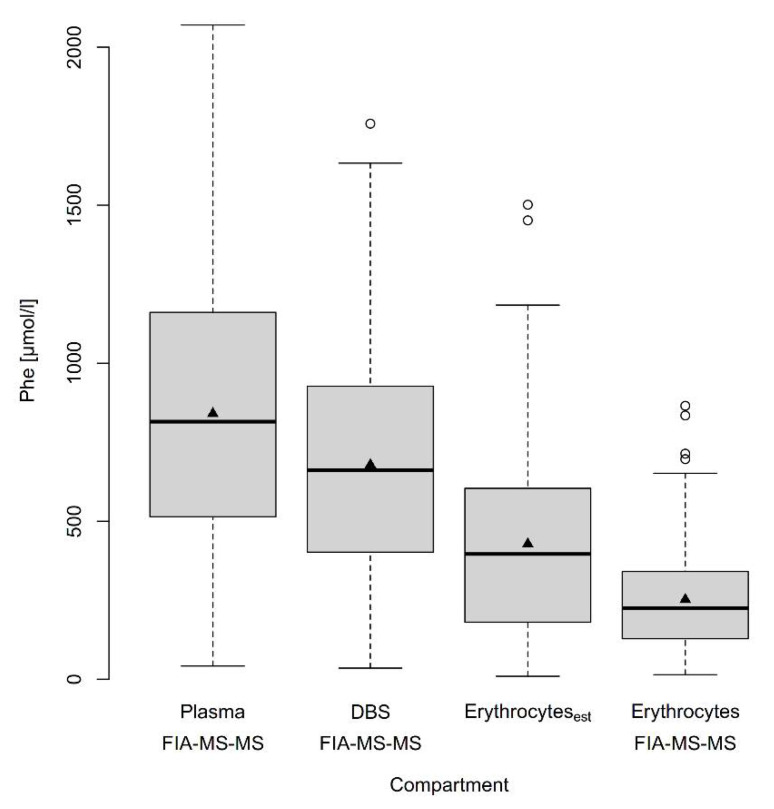
Boxplot of Phe concentrations in different blood compartments (n = 224) measured by FIA-MS-MS and of estimated Phe concentrations in erythrocytes. Boxes show interquartile ranges (25th and 75th quantiles), thick lines inside the boxes represent the median (50th quantile), and triangles the arithmetic mean. The whiskers extend to the maximum of data points not higher or lower than 1.5 times the interquartile range from the boxes. Circles indicate extreme values.

We then compared Phe concentrations in plasma, DBS and dried erythrocytes measured by the same method (FIA-MS-MS) in the 228 samples in which hematocrit (Hct) had been determined. Phe concentrations were highest in plasma (median 815, 25th quantile 514, 75th quantile 1160 µmol/L). Phe concentrations in DBS were 81% of plasma concentrations (median 661, 25th quantile 403, 75th quantile 926 µmol/L) and Phe concentrations in erythrocytes were lowest being only 28% of plasma concentrations (median 225, 25th quantile 129, 75th quantile 341 µmol/L) (Figure 3). As erythrocytes have to be washed with saline solution before analysis to avoid contamination with plasma amino acids this result may not reflect real erythrocyte Phe concentrations [10]. We therefore calculated Phe concentrations in erythrocytes using the formula suggested in this publication: Phe Erythrocytes_est_ = [(Phe whole blood − (1 − Hct) ∗ Phe Plasma)/Hct] resulting in 1.7-fold higher concentrations (median 396, 25th quantile 181, 75th quantile 604 µmol/L) (Figure 3). In four samples calculation resulted in negative values. These were excluded from further analysis resulting in n = 224 samples.

**Figure 4:**
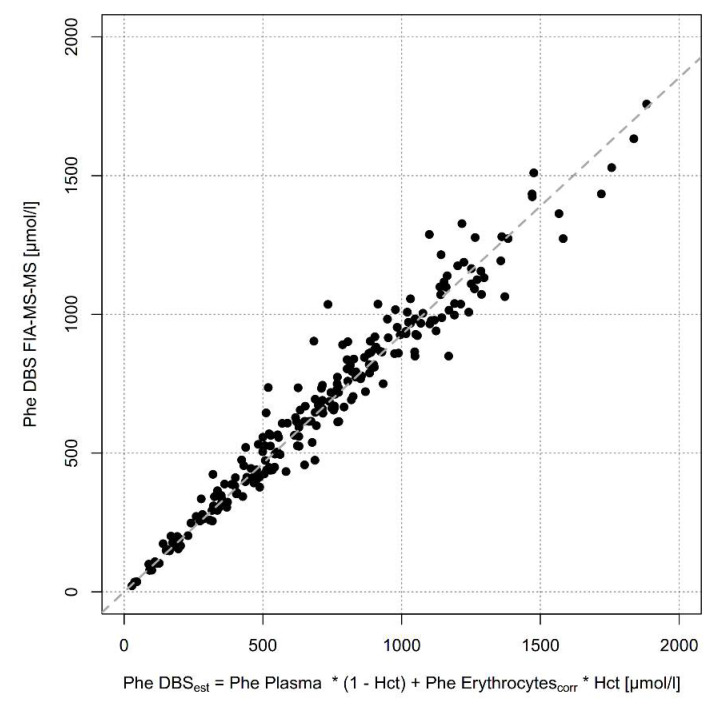
Association between estimated Phe concentrations in DBS using the formula Phe DBS_est_ = Phe Plasma ∗ (1 − Hct) + Phe Erythrocytes_corr_ ∗ Hct and Phe concentrations in DBS measured by FIA-MS-MS (n = 228). Dashed line: linear regression line.

As DBS consists of dried whole blood, i.e., a mixture of plasma and erythrocytes (as well as other cellular components), the difference between Phe concentrations in plasma and DBS is most likely caused by dilution through much lower Phe content in erythrocytes then in plasma. The proportion of erythrocytes in whole blood is specified by the hematocrit. To model this assumption, we estimated Phe concentrations in DBS by using the formula [Phe DBS_est_ = Phe Plasma ∗ (1 − Hct) + Phe Erythrocytes_corr_ ∗ Hct]. Phe Erythrocytes_corr_ was defined as the measured Phe erythrocyte concentration multiplied by 1.7, the factor of Phe lost by washout. Calculated Phe concentrations were compared with measured Phe concentrations in DBS (Figure 4) and resulted in an almost perfect good correlation of 0.93 without significant difference (adjusted R^2^ = 0.99, *p* = 0.18).

**Figure 5:**
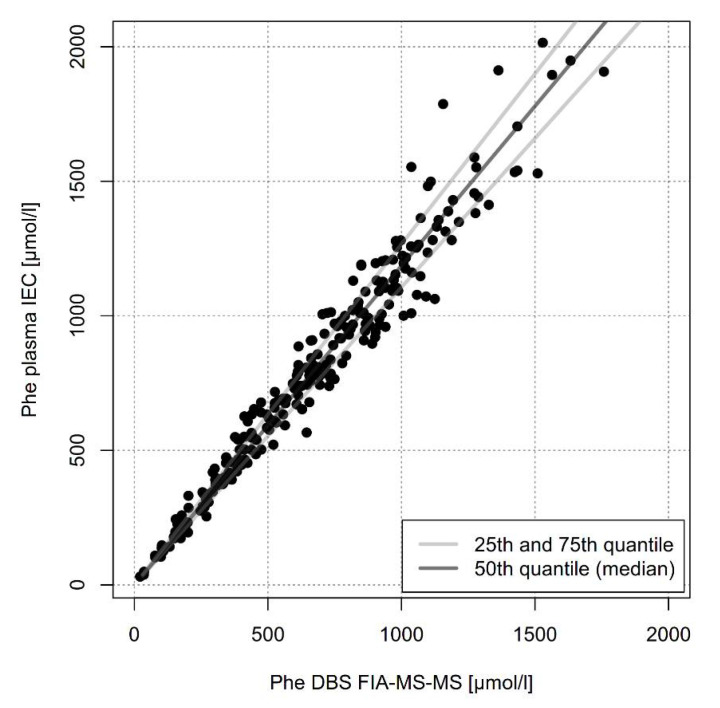
Comparison of DBS Phe concentration measured by FIA-MS-MS with plasma Phe concentration measured by IEC in 240 paired DBS and plasma specimen. The dark line represents the 50th quantile (median), the lighther lines the 25th and the 75th quantiles.

We then compared the clinically relevant sample types of DBS measured by FIA-MS-MS with plasma measured by IEC. Although there was a linear relationship (y = 1.19x) Phe plasma concentrations were about 19% higher than Phe DBS (Figure 5). However, due to heteroscedasticity, variance of predicted plasma Phe levels increases in the higher Phe concentration range.

Heteroscedasticity is present when the variability of the random disturbance is different across predicted values of the dependent variable monitored over the concentration range of the independent variable. Under this condition, estimates from linear regression are biased and can impact the validity of linear regression analysis. Significant heteroscedasticity (*p* < 0.0001) was confirmed by Breusch-Pagan test indicating increasing residuals and therefore increasing differences between Phe concentrations in plasma measured by IEC and Phe concentrations in DBS measured by FIA-MS-MS in the higher concentration range (see also Figure S1). Quantiles regression were used to model the 25th quantile (β = 1.06, *p* < 0.0001), 50th quantile (β = 1.19, *p* < 0.0001), and 75th quantile (β = 1.27, *p* < 0.0001). The 75th quantile was chosen because this percentile seems to compensate the divergence especially in the high concentration range whereas in the low concentration range deviation from the 50th quantile is only minimal. The slight overestimation of DBS Phe in the high concentration range is more acceptable than underestimation as it will result in earlier recommendation of an intensified therapy to prevent potential long-term complications.

## 3. Discussion

Monitoring of Phe concentrations in individuals with PKU is of utmost importance as therapy strategies are guided by the current values. It is also important for patients’ metabolic control that laboratory results gained by different methods are reliable and comparable. A difference between DBS and plasma phenylalanine concentrations in paired specimens has been described in several studies [4,5,7,11,12]. In these reports Phe concentration is always lower in DBS than in plasma (19–28%) which is partly attributed to “inaccuracy” of the FIA-MS-MS method [5] as well as to preanalytical factors, such as sample volume and analytical factors, such as extraction efficiency of Phe from DBS [7]. Moat et al. briefly mention possible differences between distribution of Phe in plasma and in erythrocytes [7], but do not elaborate this hypothesis.

The goal of our study was to measure Phe concentrations in different blood components from paired samples with the two methods most frequently used in clinical routine: FIA-MS-MS and IEC. FIA-MS-MS is mainly used for DBS cards sent from the patients at home and IEC is typically used for quantification of Phe and other amino acids in the outpatient clinic. To exclude preanalytical variation caused by bloodspot volume, capillary versus venous blood sampling, different test tube anticoagulants and extraction recoveries, samples were collected under controlled conditions in our outpatient clinic using venous blood either directly applied to the same kind of filter paper card for DBS analysis or collected in tubes with EDTA as anticoagulant for plasma and erythrocyte analyses.

Our data confirm a difference in Phe concentrations determined in plasma and DBS when measured by the same method (FIA-MS-MS), yielding 19% higher concentrations in plasma, which is in accordance with previously reported higher plasma Phe concentrations of 18 to 28% [4,5,11,12].

By analysis of different blood compartments by IEC and FIA-MS-MS we clearly show that the reported differences in Phe concentrations in plasma and DBS are not primarily due to the use of different laboratory methods but are caused by different Phe concentrations in plasma and erythrocytes.

We do not see a significant difference of Phe concentrations measured by IEC or FIA-MS-MS, the two most frequently used laboratory methods, when the same type of specimen is used. Analysis of 240 plasma pairs measured by IEC and FIA-MS-MS resulted in an excellent correlation with only minimal variance in a large concentration range from 0 to 2000 µmol/L. DBS pairs measured by IEC and FIA-MS-MS also showed a discrepancy of only 7% but the number of samples was much smaller (n = 10) and the concentration range was 0–1200 µmol/L. Phe determination by IEC and FIA-MS-MS in erythrocytes from the same 10 samples also showed a minor difference of only 5%.

However, Phe concentrations were significantly different in different blood components when measured by the same method. In paired samples Phe concentrations in erythrocytes (median 225 µmol/L) were less than 1/3rd of those in plasma (median 815 µmol/L). The considerably lower Phe concentration in erythrocytes could partly be caused artificially by washing Phe out of the cells during sample processing, as described in [10]. The authors show that the recovery of Phe in washed erythrocytes is only about 40% of the expected concentration when calculated by the formula: Phe Erythrocytes_est_ = [(Phe whole blood − (1 − Hct) ∗ Phe Plasma)/Hct]. They speculate that this is due to the fact that the erythrocyte transport system for Phe, methionine and the branched chain amino acids is a non-conctrating system allowing these amino acids to be washed out by saline solution. To address this problem, we calculated Phe concentrations in erythrocytes (Phe Erythrocytes_est_) by the formula above and plotted these against the measured erythrocyte concentrations. Similar to [10] the estimated erythrocyte Phe concentration was 1.7-fold higher than the measured concentration (Figure 3). The fact that the recovery described by Hagenfeldt et al. was higher than ours could be explained by the small sample numbers in [10] (n = 10).

When we compared the sample types mainly used in clinical practice, DBS measured by FIA-MS/MS and plasma measured by IEC, we saw a linear correlation with plasma Phe concentrations being 19% higher than DBS concentrations. This observation has been published before but up to now there has not been a plausible explanation for this difference. As our experiments have shown lower Phe concentrations in erythrocytes than in plasma we hypothesized that this phenomenon reflects different Phe concentrations in different blood compartments. DBS is a mixture of plasma and erythrocytes; the proportion of the latter is determined by the hematocrit. Phe concentrations in DBS are much lower than in plasma because they are influenced by low erythrocyte Phe concentration. To prove this hypothesis, we calculated Phe concentrations in DBS using the following formula: Phe DBS_est_ = [Phe Plasma ∗ (1 − Hct) + Phe Erythrocytes ∗ Hct] and compared it with measured Phe concentrations in DBS (Figure 4), and found a positive correlation of R^2^ = 0.99. When hematocrit has not been measured the formula can be simplified to Phe DBS_est_ = [(Phe Plasma + Phe Erythrocytes)/2], resulting in a good estimate in non-anemic patients beyond neonatal age. In clinical practice, however it will not be feasible to determine Phe concentrations in erythrocytes. Therefore, a reliable conversion of DBS Phe values over the whole concentration range is mandatory in clinical practice because DBS sampled at home and mailed to the lab is the most convenient and frequent method of monitoring.

Several authors have suggested to use a correction factor specific for the respective laboratory [6]. However, this approach is only valid if variance is not significantly different in various concentration ranges. We have shown that there is significant heteroscedasticity (Figure S1); therefore, predicted Phe levels from a linear regression model have doubtful validity, especially in the higher concentration range. The use of a correction factor will mainly fit to concentrations in the lower range up to 500 µmol/L but result in underestimation of high Phe concentrations. Underestimation of high Phe concentrations is problematic as it will prevent patients from intensifying their therapy. This problem could be corrected by addition of a y-axis intercept but this will lead to an overestimation of low Phe concentrations. Overestimation of Phe concentrations is especially problematic in patients treated with Pegvaliase [13,14], in whom far too low Phe concentrations leading to hair loss and skin problems commonly occur [15]. In this patient cohort the use of a correction factor plus y-axis intercept may feign higher Phe concentrations than actually present and prevent necessary actions to correct decreased Phe concentrations. Overestimation of Phe concentrations is also difficult in pregnant patients or patients planning a pregnancy, when target ranges are far lower and narrower than in adulthood resulting in the need of a much stricter diet. Falsely elevated Phe concentrations will lead to overtreatment and may result in frustration or even noncompliance. On the other hand, underestimation of very high Phe concentrations, which will occur mainly in non-compliant patients, will keep these patients from changing their regimen.

The optimal approach to convert data with distinct heteroskedasticity is quantiles regression. We suggest the slope of the 75th quantile from quantile regression as a correction factor (in our experimental setup 1.27), to cope with the fact of heteroscedasticity and therefore a larger correction of DBS Phe levels than when a correction factor is derived from simple ordinary least square regression. In the higher concentration range underestimation of DBS Phe will be more prohibited leading to earlier recommendation of a stricter diet to prevent potential long-term complications. In a given dataset of Phe concentrations in DBS and plasma the slope of the 75th quantile can be calculated by specific software programs, such as R or Gretl (open source software), as well as by commercial packages like SAS, Stata, SPSS and XLSTAT (an Excel add-on).

## 4. Materials and Methods

### 4.1. Subjects

A total of 240 samples from 199 patients (74 male, 125 female) with hyperphenylalaninemia (PKU and mild hyperphenylalaninemia) due to PAH deficiency attending our outpatient clinic for routine visits including venous blood draw were recruited for this study. All 199 patients, their parents (in minors) or guardians approved of the use of their left-over samples in an anonymized setting for method validation purposes, in agreement with institutional and national legislation and regulations for GCP (Good Clinical Practice). Patients’ mean age was 23 years (1–55). The study was approved by the ethics committee of the University Heidelberg (S-647/2017). Treatment included a Phe restricted diet only, a Phe restricted diet plus sapropterin, sapropterin only, or no treatment. No patient received Pegvaliase (Palynziq^®^). Venous blood was collected in EDTA tubes (for plasma and erythrocytes) or dripped directly on filter paper cards (for DBS). Blood spot cards (GE Whatman 903) were obtained from EBF (Eastern Business Forms, Greenville, SC, USA). All samples were analyzed in one metabolic Laboratory at the Dietmar-Hopp Metabolic Center, University Hospital Heidelberg.

### 4.2. Measurement of Phenylalanine in Dried Blood, Dried Erythrocytes and Dried Plasma Using FIA-MS/MS

Whole blood applied on filter cards was dried at least for 3 h at room temperature. Semi-quantitative determination of free Phe was performed by using the commercially available test kit MassChrom^®^ Kit for analysis of amino acids and acylcarnitines from dried blood for Newborn Screening (57,000 F, non-derivatized, Chromsystems Instrument and Chemicals GmbH, Graefelfing, Germany). Phe measurements were performed on a Waters Xevo TQD quadrupole mass spectrometer (Waters Corporation, Milford, MA, USA). The concentrations were determined using MassLynx software (Waters Corporation) to process the data and calculate the ratio of Phe sample to the Phe internal standard with stable isotope.

For determination of Phe in DBS Phe was extracted from a 3.2 mm disk punched from the filter card, which had been dried for at least 3 h at room temperature. For determination of Phe in dried plasma 5 µL of EDTA plasma were spotted on a 4.7 mm filter paper disk and dried overnight at room temperature. For the measurement of Phe in dried erythrocytes whole blood was centrifuged at 1300 g_max_ for 3 min. After washing two times with 0.9% sodium chloride, 100 µL of the erythrocytes were gently mixed with the same volume of 0.9% of sodium chloride. A total of 50 µL of the suspension was spotted on a filter paper card and dried at room temperature overnight. Phe was extracted from a 3.2 mm punch of the dried erythrocyte suspension.

Sample preparation was performed according to the method described within the MassChrom^®^ Kit. A total of 10 µL of supernatant was injected into the MS/MS system via flow-injection (FIA-MS/MS). The coefficient of variation (CV) was calculated by measuring two commercially available control samples with defined Phe concentrations (MassCheck^®^ Amino Acids, Acylcarnitines Dried Blood Spot Control, Gräfelfing/Munich, Germany). The CV of the low concentration control (124–266 µmol/L) was calculated to 7.9% (n = 96) and the high concentration control (308–692 µmol/L) was calculated to 8.2% (n = 96).

### 4.3. Measurement of Phenylalanine in Dried Blood, Dried Erythrocytes and Liquid Plasma Using Ion Exchange Chromatography (IEC)

IEC was performed on a Biochrom 30Plus amino acid analyzer using the corresponding buffers 1–6, ninhydrine (12.5%), lithium loading buffer and column (PEEK 200 mm × 4.6 mm, resin of crosslinked polymers synthesized from styrene and divenylbenzene) (Biochrom Ltd., 1020 Cambourne Business Park, Cambourne, Cambridge, CB23 6DW, UK all provided by Laborservice Onken, Gründau, Germany). For calibration, a standard with a Phe concentration of 200 µmol/L was used. The coefficient of variation (CV) was calculated by measuring ClinChek^®^ Control (10280–10282, RECIPE Chemicals + Instruments GmbH, Munich, Germany). It includes two controls with a low and a high level (Level I: 64.3–96.5 µmol/L; Level II: 360–487 µmol/L). For Control Level I and the parameter Phe a CV of 12% (n = 272) and for Control Level II and the parameter Phe a CV of 17% (n = 192) were calculated.

Five 3.2-mm spots of the dried blood or dried erythrocytes were extracted with 100 µL of lithium loading buffer for 30 min at room temperature. Next, 25 µL of standard solution (20% sulfosalicylic acid internal standards: 800 µmol/L S-(2-Aminoethyl)-L-Cysteine hydrochloride (A2636-1G, Sigma-Aldrich, Steinheim, Germany) and 800 µmol/L D-Glucosaminic acid (G0259-1G, Sigma-Aldrich, Steinheim, Germany)) were added to the extracts. For plasma analysis, 50 µL of the internal standard solution were added to 200 µL of EDTA plasma.

Samples were centrifuged at 18,620 g_max_ for 5 min, and the supernatant was diluted with lithium loading buffer (1:2; *v:v*). Next, 30 µL of the dilution were analyzed using a Biochrom 30Plus amino acid analyzer.

### 4.4. Measurement of Hematocrit

Hematocrit was measured using a standard laboratory method (Siemens Advia 2120i, Siemens Healthcare, Erlangen, Germany).

### 4.5. Statistical Analysis

R environment for statistical analysis and graphics version 4.1.0 was used for statistical analysis. Linear regression analysis was used to examine the relationship between two specimens. We used quantile regression (R package ‘quantreg’) to account for heteroscedasticity in regression models, and Breusch-Pagan test was used to test for hetero-scedasticity (R package ‘lmtest’).

## 5. Conclusions

Phe concentrations are significantly lower in erythrocytes than in plasma leading to about 19% lower DBS compared to plasma concentrations independent from the method used for quantification. By applying the slope of the 75th percentile from quantile regression as a correction factor, DBS concentrations can be reliably converted into plasma concentrations.

## Data Availability

Data are available in the article.

## Supplementary Materials

The following are available online at https://www.mdpi.com/article/10.3390/metabo11100680/s1, Table S1: Mass spectrometric parameters for the analysis of native and isotopically labelled Phe using multiple reaction monitoring (MRM) in a multiple component method after positive electrospray ionization, Figure S1: Scatterplot of residuals (difference between observed and fitted values) and fitted values from linear regression of Phe concentrations in plasma measured by IEC and Phe concentrations in DBS measured by FIA-MS-MS. With increasing fitted values, the residuals are increasing too, indicating heteroscedasticity (*p* < 0.001; Breusch Pagan test).
