# Supplementary material for: Differences of Phenylalanine Concentrations in Dried Blood Spots and in Plasma: Erythrocytes as a Neglected Component for This Observation
Source: Metabolites. 2021 Oct 3;11(10):680. doi: 10.3390/metabo11100680 (PMC8537883; doi:10.3390/metabo11100680)

Table S1: Mass spectrometric parameters for the analysis of native and isotopically labelled Phe using multiple reaction monitoring (MRM) in a multiple component method after positive electrospray ionization.

Parameter	Value
**Mass spectrometer**	
Capillary voltage (kV)	4
Cone voltage (V)	24
Collision energy (V)	14
Desolvation Gas Flow (L/h)	650
Cone Gas Flow (L/h)	50
Desolvation Temperature (C°)	420
Dwell times (s)	0.01
**Analyte mass transitions**	
Phenylalanine	165.9 Da → 120 Da
Phenylalanine internal standard	170.9 Da → 125 Da

Figure S1. Scatterplot of residuals (difference between observed and fitted values) and fitted values from linear regression of Phe concentrations in plasma measured by IEC and Phe concentrations in DBS measured by FIA-MS-MS. With increasing fitted values, the residuals are increasing too, indicating heteroscedasticity ($p < 0.001$; Breusch Pagan test).

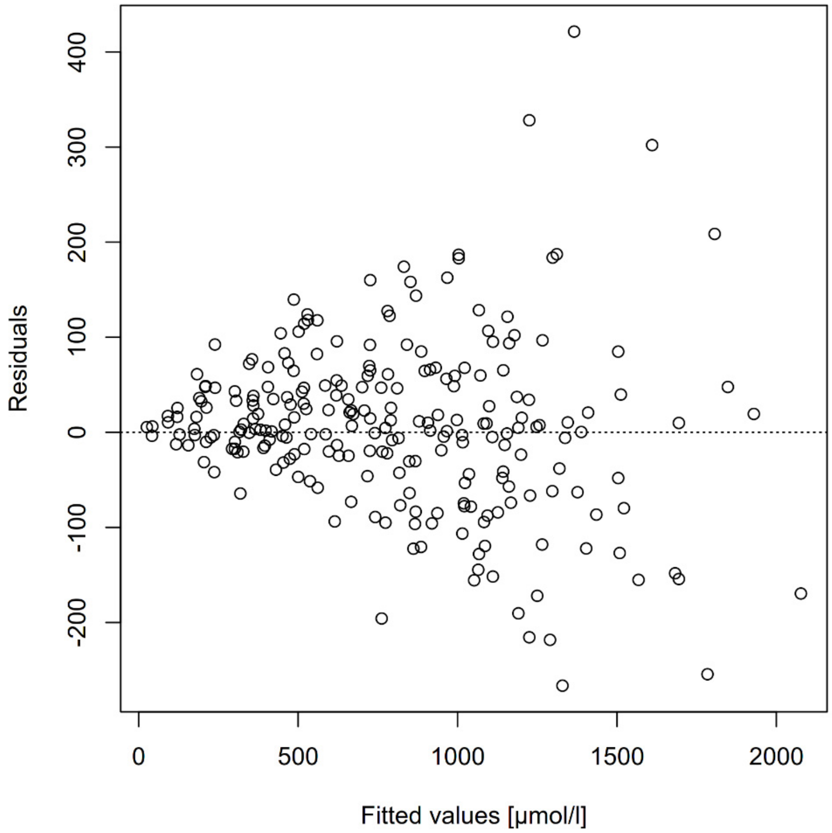

Supplement: Supplementary file 1 [file metabolites-11-00680-s001.zip › metabolites-1399788-supplementary.pdf]
